# Layer-by-layer assembly of CsPbX_3_ nanocrystals into large-scale homostructures†

**DOI:** 10.1039/d2nr04169c

**Published:** 2022-10-07

**Authors:** Matilde Cirignano, Sergio Fiorito, Matteo Barelli, Vincenzo Aglieri, Manuela De Franco, Houman Bahmani Jalali, Andrea Toma, Francesco Di Stasio

**Affiliations:** Dipartimento di Chimica e Chimica Industriale, Università, Degli Studi di Genova Via Dodecaneso 31 16146 Genoa Italy; Photonic Nanomaterials, Istituto Italiano di Tecnologia Via Morego 30 16163 Genoa Italy francesco.distasio@iit.it; Clean Room Facility, Istituto Italiano di Tecnologia Via Morego 30 16163 Genoa Italy; Nanochemistry, Istituto Italiano di Tecnologia Via Morego 30 16163 Genoa Italy

## Abstract

Advances in surface chemistry of CsPbX_3_ (where X = Cl, Br or I) nanocrystals (NCs) enabled the replacement of native chain ligands in solution. However, there are few reports on ligand exchange carried out on CsPbX_3_ NC thin films. Solid-state ligand exchange can improve the photoluminescence quantum yield (PLQY) of the film and promote a change in solubility of the solid surface, thus enabling multiple depositions of subsequent nanocrystal layers. Fine control of nanocrystal film thickness is of importance for light-emitting diodes (LEDs), solar cells and lasers alike. The thickness of the emissive material film is crucial to assure the copious recombination of charges injected into a LED, resulting in bright electroluminescence. Similarly, solar cell performance is determined by the amount of absorbed light, and hence the light absorber content in the device. In this study, we demonstrate a layer-by-layer (LbL) assembly method that results in high quality films, whose thicknesses can be finely controlled. In the solid state, we replaced oleic acid and oleylamine ligands with didodecyldimethylammonium bromide or ammonium thiocyanate that enhance the PLQY of the film. The exchange is carried out through a spin-coating technique, using solvents with strategic polarity to avoid NC dissolution or damage. Exploiting this technique, the deposition of various layers results in considerable thickening of films as proven by atomic force microscope measurements. The ease of handling of our combined process (*i.e.* ligand exchange and layer-by-layer deposition) enables thickness control over CsPbX_3_ NC films with applicability to other perovskite nanomaterials paving the way for a large variety of layer permutations.

## Abstract 2

Layer-by-layer fabrication of CsPbX_3_ (where X = Cl, Br or I) nanocrystal films enabled by solid-state ligand exchange.

## Graphical abstract

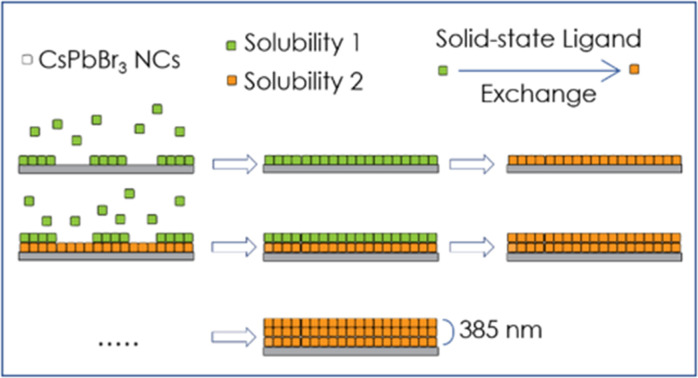

## Introduction

Metal halide perovskites (MHPs) have quickly gained prominence in optoelectronics thanks to their exceptional optical properties^1^ and the possibility to fabricate low-cost, large-area and light-weight devices.^2^ Among the vast family of MHP materials, colloidal nanocrystals (NCs) with the composition CsPbX_3_ (where X = Cl, Br or I), FAPbX_3_ and MAPbX_3_ (where FA = formamidium, MA = methylammonium) have been exploited already in many applications^3,4^ such as lasing,^5,6^ light-emitting diodes (LEDs),^7–9^ single photon sources,^10^ photodetectors,^11,12^ photovoltaics,^13,14^ photocatalysis,^15,16,20^ and as either singlet or triplet sensitizers.^17–19^ MHP NCs show size- and composition dependent optical properties,^21^ while their bandgap can be tuned from the blue to the near-infrared spectral region.^6^ One of the main advantages of perovskite NCs is their facile synthesis procedure compared to II–IV^22^ and III–V^23^ NCs leading to near-unity photoluminescence quantum yield (PLQY) in solution.^24^ Importantly, the performance of LEDs based on MHP NCs directly depends on the PLQY of the latter. However, MHP NCs are embedded in LEDs as thin films and the PLQY value is typically diminished moving from solution to the solid state^24^ due to the high density packing of NCs leading to energy-transfer to trap-states and increased self-absorption.^25^ Native surface ligands (such as oleate and oleylammonium) that typically cover the NCs in solution do not provide effective surface passivation in films, in particular if they are exposed to ambient air and/or moisture.^1^ Also, such long carbon chain ligands can disrupt charge transport and hinder the application of MHP NCs in solar cells and photodetectors.^26^ Consequently, several efforts have been dedicated to optimize the quality of MHP NC films.^27–29^ Ligand exchange (*i.e.* the substitution of the organic molecules covering the NC surface with other molecules) is exploited to tackle the aforementioned issues,^30^ adjust dispersibility in varying solvents, prepare NCs for their use in different applications^31–34^ or add ligand functional groups that could not be included during the direct synthetic process.^35,36^ Ligand exchange has been demonstrated to be an effective strategy to improve the electrical conductivity of NC-based films for device applications^37,38^ by applying shorter ligands that facilitate the transfer of photo-excited charges.^39^ A very successful approach is the ligand exchange with didodecyldimethylammonium^+^ (DDA^+^),^31^ which has been proven to have a strong affinity to negative sites^32^ and it leads to improved stability and PLQY.^40,41^ Another ligand that has been used to achieve MHP NC solutions with near-unity PLQY is thiocyanate (SCN^−^),^42^ which replaces 10–15% of the negative surface atoms thus removing shallow traps.^42^ The overall improvement in performance induced by ligand exchange procedures is also translated in optoelectronic devices. For example, Zheng *et al.* demonstrated an external quantum efficiency (EQE) of 13.4% for CsPbBr_3_ NC LEDs treated with didodecyldimethylammonium bromide^43^ while Chen *et al.* in 2019 fabricated the first device based on CsPbBr_3_ NCs treated with ammonium thiocyanate.^44^ All these treatments have been carried out in the liquid phase through quick and simple procedures of mixing NC solutions with an excess of the new ligands, but it would be of great interest to assess if similar ligand exchange procedures can be successfully performed directly in solid-state films. In fact, ligand exchange in the solid state can promote increased PLQY and stability, and it can enable the fabrication of films *via* layer-by-layer (LbL) assembly, as commonly used for PbS NCs.^45,46^ The LbL assembly has been developed in the past few years and adapted for specific use in very different fields.^47^ In a standard LbL assembly procedure used for NC films, a first layer of NCs is deposited from the colloidal solution and then the polarity of the surface is switched *via* ligand exchange. This technique allows the deposition and processing of each new NC layer without affecting the underlying ones.^13,48,49^ Progressively constructing films with thin layers of NCs allows for fine control over the total emissive material thickness, while imparting different functionalities.^45,46^ To the best of our knowledge, such a concept has not been successfully demonstrated for MHP NCs since perovskite NCs are prone to PL quenching and damage when exposed to polar solvents.^50^ Here, we developed a LbL approach based on solid-state ligand exchange on CsPbBr_3_ NC films. Using this method, we were able to increase the thickness of the film while maintaining homogeneous drafting, NC shape, and crystal structure and providing a near-unity in-film PLQY. In this study, we focused on two of the most widely used exchanging ligands: DDAB^31^ and NH_4_SCN.^42^ Indeed, both ligands are well known to promote high PLQY and improved stability. The processing of both ligands was precisely optimized to avoid any damage of the NCs or the homogeneity of the film. Ligand solutions were then softly dynamic-cast onto the perovskite layer. Through this method, we obtained CsPbBr_3_ NC films with a thickness up to 385 nm and emitting at 511 nm with a PLQY approaching 100%. Finally, we also demonstrated that our protocol can be extended to other emissive MHP NCs such as CsPbI_3_.

## Results and discussion

**Fig. 1:**
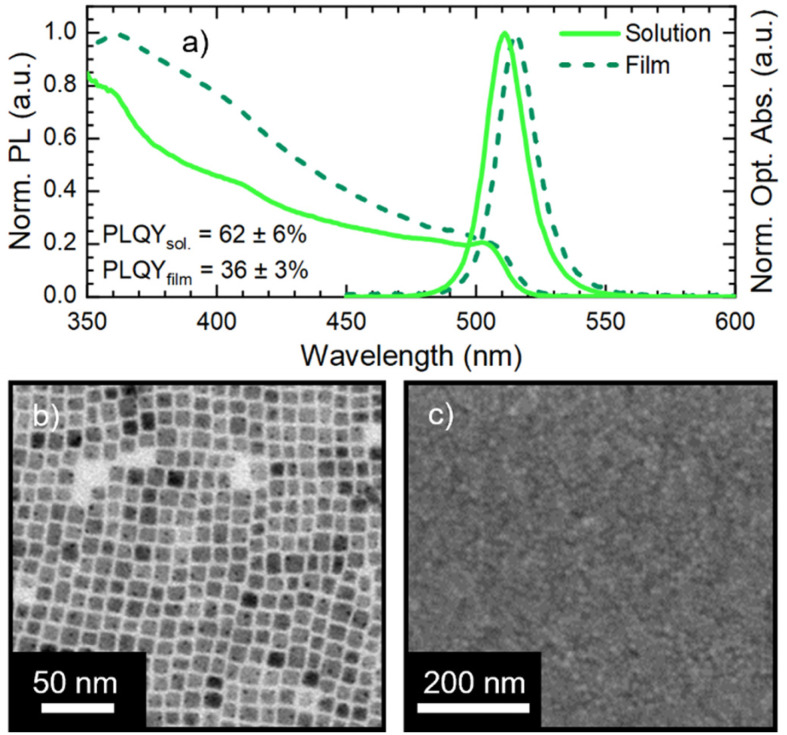
(a) Normalized optical absorption and PL of pristine CsPbBr_3_ nanocrystals in solution (solid line) and in the film (dashed line). (b) Representative TEM micrograph of pristine CsPbBr_3_ nanocrystals. (c) Representative SEM micrograph of CsPbBr_3_ nanocrystal layer on ITO substrate.

We synthesized CsPbBr_3_ NCs stabilized with oleic acid (OA) and oleylamine (OLA) ligands *via* hot-injection synthesis reported by Baranov *et al.*^51^ with some modifications (see the Experimental section in the ESI†). The as-synthesized NC solution shows an absorption peak and a photoluminescence (PL) peak at 504 nm and 511 nm, respectively (Fig. 1a) with a PL full-width-at-half-maximum (FWHM) of 18 nm. The obtained NCs have a cubic shape with a lateral size of 8 ± 2 nm (Fig. 1b, size distribution in Fig. S1†). We used CsPbBr_3_ NCs for the fabrication of films *via* spin coating on an ITO (indium tin oxide)/glass substrate. The CsPbBr_3_ NC solutions used in film fabrication had a Pb concentration of 1.30 ± 0.03 mg ml^−1^. Pristine CsPbBr_3_ NC films show only a slight modification of the optical absorption with respect to the starting solution, while the PL peak red-shifts to 515 nm (Fig. 1a). The 4 nm PL red-shift is due to the NC close packing in the film, which induces energy transfer from small to large NCs and increases self-absorption.^24^ The SEM micrograph in Fig. 1c demonstrates that the film obtained from pristine CsPbBr_3_ NCs is compact and uniform. Finally, the pristine CsPbBr_3_ NCs show a PLQY of 62 ± 6% in solution and 36 ± 3% in the film. The PLQY drop transitioning from solution to the film is in agreement with the literature.^52^

**Fig. 2:**
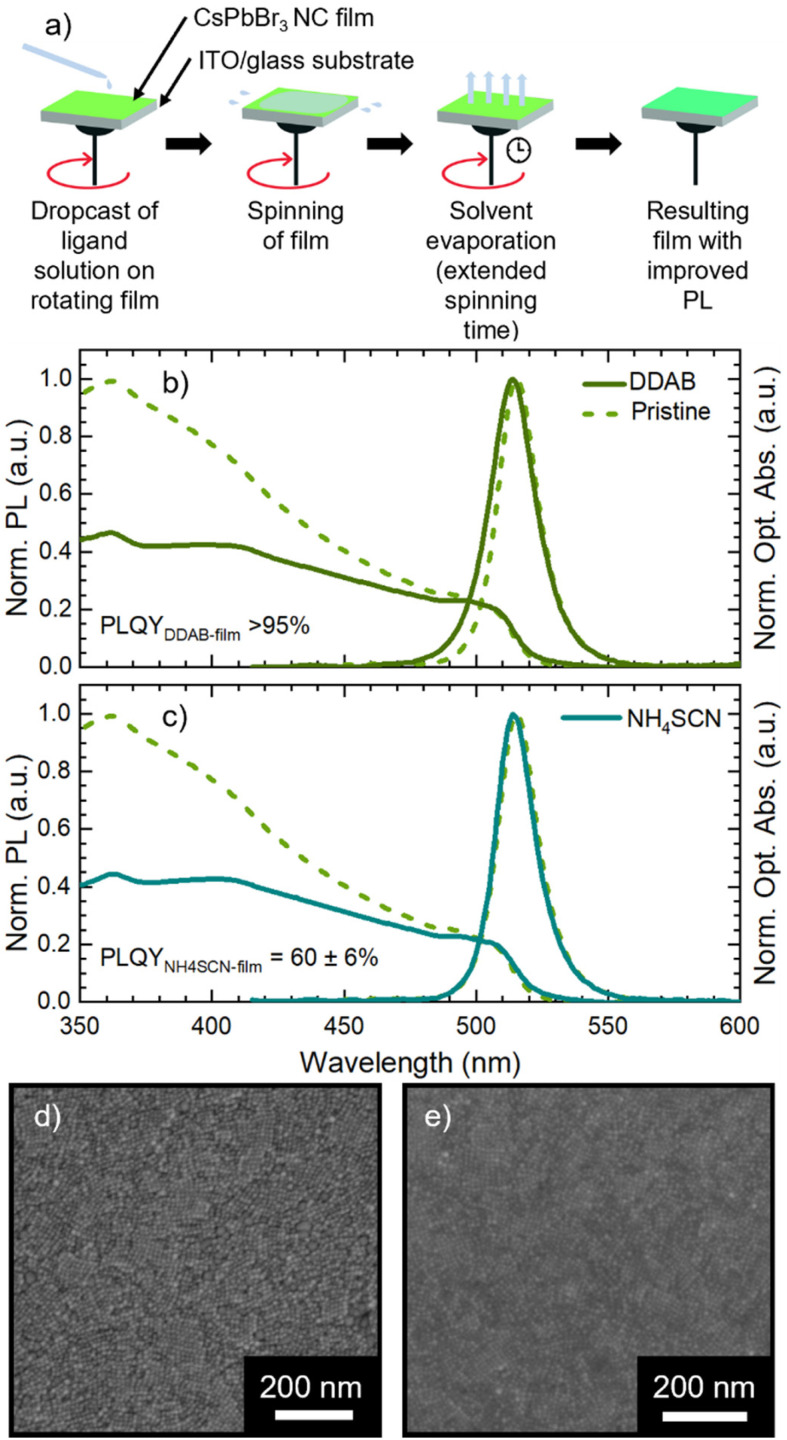
(a) Scheme of solid-state ligand exchange performed on spin-coater; (b) normalized optical absorption and PL of DDAB solid state treated film (solid line) and of pristine film (dashed line); (c) normalized optical absorption and PL of NH_4_SCN solid state treated film (solid line) and of pristine film (dashed line). (d) SEM micrograph of a DDAB solid state treated film on ITO/glass substrate. (e) SEM micrograph of a NH_4_SCN solid state treated film on ITO/glass substrate.

The pristine CsPbBr_3_ NC films were employed for assessing the impact of ligand exchange on the film and the development of the LbL assembly. We selected two ligands with a demonstrated beneficial impact on MHP NC performance: DDAB^31^ and NH_4_SCN.^42^ The typical ligand treatment in the film is fully performed on the spin-coater in air (Fig. 2a): firstly, the deposition of CsPbBr_3_ NC concentrated solution is carried out in static mode followed by rotation (standard film fabrication, 1500 rpm for 40 seconds). Afterward, the ligand treatment procedure is performed in dynamic mode (or spin-cast, 1500 rpm for 120 seconds): the ligand solution is dropped on the rotating substrate and left in movement for an extended time.

Most of the times, solution-state ligand exchange involves the addition of the replacing ligand to the NC solution. In contrast, to carry out the procedure on a film, other than avoiding polar solvents that damage the NCs, attention must also be paid to the use of nonpolar solvents that can dissolve the deposited film. Therefore, tuning the polarity of the solvent used for ligand exchange in the film has a crucial role in avoiding both the dissolution of the already deposited NC film and their damage. The solvent must also be able to dissolve the new ligand and one must consider that the concentration in solution of the latter is very important since it must be adapted to the amount of NCs deposited on the substrate. Indeed, an excessive amount of ligands in the solution for treating the film can damage the NCs and induce PL quenching. Instead, a low concentration of new molecules in the exchange solution does not lead to modification of the solubility of the NCs, thus preventing the LbL assembly.

All these considerations induced us to carry out a comprehensive study (see Table 1) in which we analyzed the effect of many different solvents with varying ligand concentrations. For each solvent, we made three different tests through spin-casting: firstly, we spin-cast the solvent on a pristine NC film to check the preservation of the homogeneity of the film and the variation of the PLQY. For those solvents that succeeded in the first trial, we continued with two additional tests employing DDAB and NH_4_SCN solutions based on the same solvent. When the right concentration of the ligand (able to preserve film homogeneity) was found, success of the ligand exchange procedure was evaluated through PLQY measurements. We found that it is not possible to completely preserve the PLQY when only solvents (*i.e.*, without ligands dissolved into them) are spun on the surface of the pre-deposited films. This is due to a localized field generated by the solvent dropped (*i.e.*, polar solvent) on the film, as discussed by Choi *et al.*^53^ Nevertheless, in some cases, we noticed an increase in the PLQY after the spin-casting of the solutions containing also the new ligands.^54^ Through our comprehensive investigation, we determined that the best performing solvent for treatment is methyl acetate for CsPbBr_3_ NCs (relative polarity of 0.253 in a scale where water polarity is 1). In fact, ligand exchanged films with methyl acetate as solvent demonstrated a relative improvement in the PLQY of 263% with DDAB. For comparison, the second-best solvent is ethyl benzoate, and it induces an improvement of the PLQY of only 34% with DDAB. Another interesting candidate is anisole, which shows a PLQY improvement of 31% with DDAB. As reported for ligand exchange carried out in solution for both SCN^42^ and DDAB,^31^ the improvement in the PLQY can be ascribed to the enhanced passivation of the NC surface which in turn decreases the defect density able to quench the photoluminescence. A similar effect is at play here, when carrying the ligand-exchange in the solid state. Apart from methyl acetate, our data confirms the existence of a polarity range (0.327–0.198) with a decreased probability of damaging the NCs. This range consists of solvents such as 2-butanone, dichloromethane, and ethyl acetate. The improved performance of methyl acetate as a solvent could be ascribed to the hydrolysis taking place in the presence of air.^13,49^ Hydrolysis of esters (*e.g.*, methyl acetate, ethyl acetate and ethyl benzoate) is more favored than that of ketones (2-butanone) and of dichloromethane due to the presence of electron-attracting groups stabilizing the electrophilic carbon. Therefore, MeOAc will be more likely to react with water in the air and, consequently, it will also remove more ligands among the solvents considered (hypothesis which is further sustained by the greater loss of PLQY when MeOAc is used alone, *i.e.*, without ligands dissolved in it). However, the greater removal of ligands will allow for more new ligands to attach to the NC surface, thus leading to a stronger increase in the PLQY. It is well known that CsPbX_3_ is more ionic in nature compared to other types of NCs (*i.e.*, II–IV and III–V NCs);^23,24^ therefore, interactions between NCs and capping ligands have a more ionic nature as well and they are susceptible to be supplanted by polar solvents. Clearly, in the previously mentioned polarity range, this effect is minimized (Table 1).

**Table 1:** Solvent tested (column 1), relative polarity (column 2) of solvents tested for solid-state ligand treatment; film response in terms of PLQY for spin-casting of solvent only (column 3); film response in terms of PLQY for spin-casting of DDAB solution (column 4) and NH_4_SCN solution (column 5)

Solvent	Relative polarity	Solvent only	Solvent + DDAB	Solvent + SCN
Water	1	Damaged film	—	—
2-Dichloropropan	1	Damaged film	—	—
Ethylene glycol	0.79	Damaged film	—	—
Methanol	0.762	Damaged film	—	—
Acetic acid	0.648	Damaged film	—	—
1-Propanol	0.617	Damaged film	—	—
Isopropanol	0.546	Damaged film	—	—
1,2-Dichloroethane	0.327	Damaged film	—	—
2-Butanone	0.327	Preserved film	↑13% PLQY	—
		↓30% PLQY		
Dichloromethane	0.309	Preserved film	Damaged film	NH_4_SCN not dissolved
Chloroform	0.259	Damaged film	—	—
Methyl acetate	0.253	Preserved film	Preserved film	Preserved film
		↓54% PLQY	↑263% PLQY	↑40% PLQY
Diglyme	0.244	Damaged film	—	—
Ethyl acetate	0.228	Preserved film	Damaged film	Preserved film
		↓24% PLQY		↑8% PLQY
Ethyl benzoate	0.228	Preserved film	Preserved film	Preserved film
		↓50% PLQY	↑34% PLQY	↓21% PLQY
Anisole	0.198	Preserved film	Preserved film	—
			↑31% PLQY	
*m*-Xylene	0.178	Damaged film	—	—
1,4-Dioxane	0.164	Damaged film	—	—

**Fig. 3:**
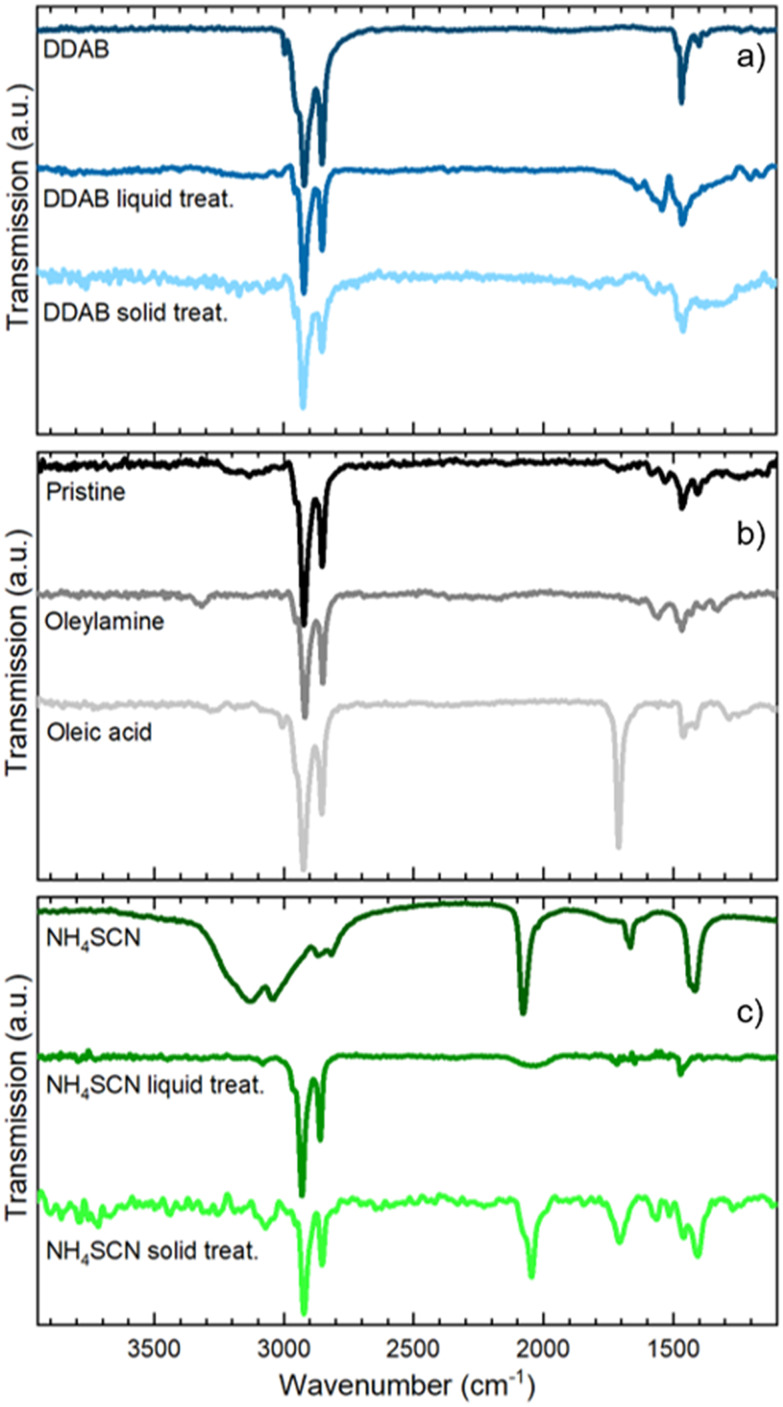
(a) FTIR spectra of DDAB bare molecule, DDAB treated NCs in solution and treated film; (b) FTIR spectra of pristine NC film, oleylamine and oleic acid bare molecules; (c) FTIR spectra of NH_4_SCN bare molecule, NH_4_SCN treated NCs in solution and treated film.

**Fig. 4:**
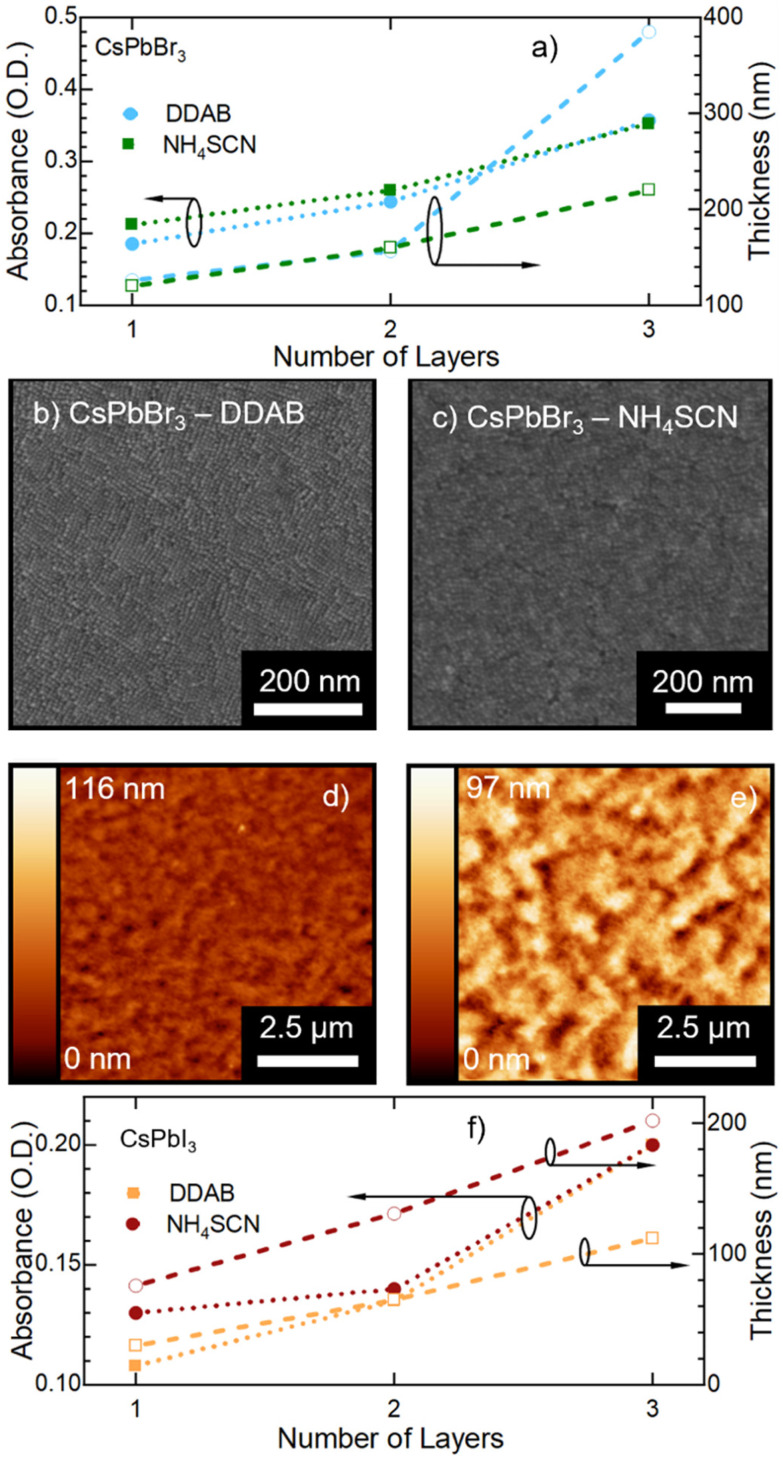
(a) Optical density of CsPbBr_3_ nanocrystal films treated with DDAB, NH_4_SCN and deposited *via* layer-by-layer method and thicknesses (lines are a guide to the eye) with increasing number of layers. (b) SEM picture of the second layer built by the LbL method (DDAB) (c) SEM picture of the second layer built by the LbL method (NH_4_SCN). (d) AFM topography of the second layer built by the LbL method, using DDAB and (e) NH_4_SCN, the RMS roughness reads 9 and 21 nm respectively; (f) optical density of CsPbI_3_ nanocrystal films treated with DDAB, NH_4_SCN and deposited *via* the layer-by-layer method and thicknesses (lines are a guide to the eye) with increasing number of layers.

In Fig. 2b and c we present the optical and morphological characterization of DDAB and NH_4_SCN treated films *via* methyl acetate solutions, respectively. As previously introduced, both ligand exchanged films show a considerable increase in PLQY from 36 ± 3% for pristine films to >95% for DDAB and 60 ± 6% for NH_4_SCN exchanged ones, respectively. The difference in the PLQY enhancement between the two ligands can be ascribed to the different concentrations used. In fact, SCN polarity causes degradation of the NCs if an excessive amount is employed during the ligand-exchange process. Consequently, the concentration of SCN is limited compared to that of DDAB during the exchange and this could imply a reduced number of molecules exchanged and therefore a contained increase in the PLQY. For comparison, we carried out ligand exchange with the two molecules in solution (see the ESI, Fig. S1 and S2†) and we measured the PLQY of the resulting films (fabricated after the exchange). In this case, we measured a PLQY of 92 ± 8% and 40 ± 4% for spin-coated films obtained from CsPbBr_3_ NCs ligand-exchanged in solution with DDAB and NH_4_SCN, respectively. Surprisingly, these findings suggest that the ligand exchange procedures carried out directly on the film have been even more impactful than in solution. We do not observe any substantial PL shift in the solid state treated films (Fig. 2b and c) with respect to the pristine film; similarly the FWHM is not strongly affected and neither is the optical absorption. The lack of changes in the PL spectra indicates that the NCs do not change their size following the ligand exchange procedure. This observation is further confirmed by the SEM imaging of both films (see Fig. 2d and e for DDAB and NH_4_SCN, respectively) where we observe that the films remain uniform, and the NC morphology is unchanged. To shed light on the nature of the ligand exchange procedure, we performed FTIR (Fourier-transform infrared spectroscopy) measurements on pristine and exchanged samples (both in solution and in the film). In the DDAB treated samples (see Fig. 3a), the overlap between the peaks of DDAB and oleylamine (Fig. 3b) at 1465 cm^−1^, caused by a very similar molecular structure, makes the presence of DDAB not striking. However, a similar modification of the peak placed at 1480 cm^−1^ (corresponding to CH_2_ bending) is noticeable in both the liquid and solid treated samples. In the NH_4_SCN treated samples shown in Fig. 3c, the 2082 cm^−1^ peak indicates the presence of thiocyanate as demonstrated by the spectrum of the bare molecule used for the treatment. The region around this frequency is typically related to the presence of ^−^SC≡N. Noteworthily, 2048 and 1400 cm^−1^ peaks appear with a slight shift (40 cm^−1^) with respect to the NH_4_SCN free molecules. This can be ascribed to the different atom surroundings, which are attributed to the binding of the ligand to the NC surface. We also observe a peak at 1704 cm^−1^ corresponding to oleic acid molecules (Fig. 3b), indicating that footprints of native ligands are still present both in solution and in the film, even if in different intensities. Moreover, in the NH_4_SCN treated film, peaks are more similar in intensity to the C–H attributed ones (2926 cm^−1^ 2856 cm^−1^) with respect to the liquid treated sample. From these observations, we can deduce that the ligand exchange procedures do not involve all the original ligands but only a given amount that is sufficient to improve the optical properties of the material. This explanation is further supported by the PL spectra of the treated films (Fig. 2b and c), where only minor modifications are observed. When comparing the exchange in solution and film we can conclude that for DDAB solution treatment, new peaks are more evident with respect to the solid phase treatment, as expected considering the amount of surface exposed to new ligands. For the NH_4_SCN treatment, we noticed more intense peaks related to the new ligand in the solid phase treatment which can be ascribed to the ease of filling vacancies on the NC surface given the small size of the molecule. After studying and optimizing the solid-state ligand treatment, we moved to perform the LbL-assembly using spin-coating (also known as “spin assembly”) with the same rotating speed used for film fabrication. Among the few other methods available for multilayer film fabrication,^55^ we decided to use spin-coating for its convenience and speed of process. As an example, comparing this technique to immersive methods of multilayer deposition, spin-coating quickens the assembly process significantly, allowing for layers to be deposited in ∼30/40 seconds due to the various forces governing it.^56^ These forces include electrostatic interactions, centrifugal force and so on, which allows the spin assembly to be much faster than immersive procedures. Furthermore, spin assembly typically produces more homogeneous films.^47^ The solid-state ligand treatment changes the solubility of the deposited NC layer and allows the deposition of subsequent layers. Yet, we must consider the solubility of the new ligands to decide which solvent we can employ to deposit additional NC layers. For example, DDAB is soluble in toluene, thus it would be counterproductive to use toluene for the second NC layer. However, DDAB is not soluble in octane at room temperature, therefore we employed the latter solvent for the LbL assembly. Instead, NH_4_SCN is insoluble in toluene. The deposition of additional layers can be repeated various times until the desired thickness is achieved. The resultant CsPbBr_3_ NC films exhibit an increase in optical absoprtion with the increasing layer number for both DDAB and NH_4_SCN, indicating uniform surface morphologies (Fig. S3†). Optical density (OD) values of the absorption peak and relative thickness of each layer of the emissive film built *via* LbL assembly after the exchange with NH_4_SCN and DDAB are shown is Fig. 4a (line between symbols are a guide to the eye). Similarly, the film thickness increases upon deposition of subsequent layers, as demonstrated by AFM thickness analysis (Fig. 4a). SEM pictures of CsPbBr_3_ NCs film after LbL assembly are shown in Fig. 4b and c (DDAB and NH_4_SCN respectively) and uniformity, shape and size of nanocrystals are preserved. Upon the deposition of 3 layers, we were able to increase the average film thickness up to 385 nm for DDAB and 220 nm for NH_4_SCN treated samples (see atomic force microscopy data reported in Table S1,†Fig. 4d and e, and Fig. S4†). Importantly, the increased thickness is coupled with the high PLQY induced by the ligand exchange procedure (>95% for DDAB and 60 ± 6% for NH_4_SCN, respectively) and a limited RMS roughness of the resulting thick film: 28 nm for DDAB and 11 nm for NH_4_SCN, respectively (see Table S1†).

Finally, we demonstrate that the LbL assembly method we developed is applicable to other MHP NCs as well by adapting the polarity of the used solvent. For instance, we applied our LbL assembly to CsPbI_3_ NCs dispersed in hexane or octane with analogous ligand solutions of DDAB and NH_4_SCN. Yet, in this case we selected ethyl acetate as the solvent for the solid-state ligand exchange, as methyl acetate was causing decomposition of CsPbI_3_ into lead depleted phases^57^ (relative polarity of 0.228 for ethyl acetate *vs.* 0.253 for methyl acetate). We were able to obtain a final CsPbI_3_ NC film with a thickness of up to 200 nm for a DDAB treated sample (Fig. 4f, Fig. S6 and S7†). It is noticed that an increase of the optical density with the number of layers is consistent with a thickening of the emissive material. In the case of the NH_4_SCN ligand treatment, the optical density decreases after the third layer, possibly indicating degradation of the film. Importantly, future extensive testing of other ligands and ligand-concentrations can further improve the LbL processing of CsPbI_3_ films bringing it on-par with CsPbBr_3_. Here, we focused on demonstrating that “ad-hoc” tailoring of our process has the potential for application on other perovskite NC compositions.

## Conclusions

In this work, we introduced a solid-state ligand exchange of perovskite NC films followed by layer-by-layer assembly based on spin-coating. Through the introduction of didodecyldimethylammonium bromide and ammonium thiocyanate as new ligands on the NC surface, we achieved an improvement in the PLQY value of CsPbBr_3_ NC films: >95% for DDAB and 60 ± 6% for NH_4_SCN. A comprehensive study of the different solvents that can be used in the ligand exchange procedure was carried out leading to the determination of a polarity range where polar interaction with NCs is minimized (0.327–0.198). Thanks to this study, we found that methyl acetate is the best solvent to perform solid-state ligand treatment on CsPbBr_3_ NCs with both ligands tested. We verified the successful insertion of the new ligands through FTIR measurements. Also, AFM and optical density analysis demonstrated the trend of increasing film thickness with the increasing number of depositions (*i.e.* layer-by-layer assembly of homostructures), which are enabled by a change in the solubility induced by the new surface properties. We were able to fabricate homogeneous films with a thickness of up to 385 nm. Finally, we demonstrated the versatility of our method by applying it to CsPbI_3_ NC films, thus paving the way for the application of our approach in different types of perovskite NCs.

## Author contributions

The manuscript was written through contributions of all authors. All authors have given approval to the final version of the manuscript.

## Conflicts of interest

The authors have no conflicts to declare.

## Supplementary Material

NR-014-D2NR04169C-s001
