# Supplementary material for: Layer-by-layer assembly of CsPbX3 nanocrystals into large-scale homostructures
Source: Nanoscale. 2022 Oct 7;14(41):15525–32. doi: 10.1039/d2nr04169c (PMC9612634; doi:10.1039/d2nr04169c)
Supplement: NR-014-D2NR04169C-s001 [file NR-014-D2NR04169C-s001.pdf]

Electronic Supplementary Material (ESI) for Nanoscale.
This journal is © The Royal Society of Chemistry 2022

Electronic Supplementary Information

Layer-by-Layer Assembly of $CsPbX_3$ Nanocrystals into large scale homostructures

Matilde Cirignano[a,b], Sergio Fiorito[b], Matteo Barelli[b], Vincenzo Aglieri[c], Manuela De Franco[a,b], Houman Bahmani Jalali[b,d], Andrea Toma[c] and Francesco Di Stasio[b].*

a) Dipartimento di Chimica e Chimica Industriale, Università, Degli Studi di Genova, Via Dodecaneso 31, 16146, Genoa, Italy

b) Photonic Nanomaterials, Istituto Italiano di Tecnologia, Via Morego 30, 16163, Genoa, Italy

c) Clean Room Facility, Istituto Italiano di Tecnologia, Via Morego 30, 16163, Genoa, Italy

d) Nanochemistry, Istituto Italiano di Tecnologia, Via Morego 30, 16163, Genoa, Italy

EXPERIMENTAL SECTION.

Chemicals. Lead Bromide ($PbBr_2$, 99,99%), Lead Iodide (PbI_2, 99,99%), Cesium Carbonate (Cs_2CO_3, reagent Plus, 99%), Hydroiodic acid (HI, 57%), Methyl Acetate (MeOAc 98.8%), Ethyl Acetate (EtOAc 99,5%), Toluene (anhydrous, 99.5%), 1-Octadecene (ODE, technical grade, 90%), Oleylamine (OLAm, technical grade 70%), Oleic Acid (OA, 90%), Didodecyldimethylammonium Bromide (DDAB, 98%), Ammonium Thiocyanate (NH_4SCN, ≥97.5%). All chemicals were purchased from Sigma-Aldrich and used without further purification.

Cesium oleate precursor. 400 mg of cesium carbonate were dissolved in 1.75 ml of oleic acid and 15 ml of octadecene inside a 40 ml glass vial heated to 100-120 ºC under the flow of nitrogen; the reaction was considered complete once all visible solid disappeared. The precursor was cooled down to room temperature under stirring, turning cloudy white. That room temperature cloudy suspension was used for subsequent injections with preheating.

Synthesis of $CsPbBr_3$ Nanocubes. NCs were synthesized via hot injection (165°C) of a cesium oleate precursor (500 uL) into a lead bromide (72 ± 2 mg) solution in 1-octadecene (ODE) (5mL) in presence of surfactants: oleic acid (50 uL) and oleylamine (500 uL). The injection was carried out during cooling towards room temperature under stirring (no ice bath). When the solution reached 30°C, it was transferred in 4 vials and centrifuged at 4000 rpm for 3 minutes. Supernatant was discarded and the walls of the vials dried with a swab to remove excess of solvent. Then, precipitate was centrifuged once more at 4000 rpm for 1 minute and dried again. Finally, precipitate was redispersed in 1 mL of toluene for each vial.

Preparation of Stock Solution of RNH_3I (OLA-HI). 10 ml of OLA and 1 ml of HI were loaded in a 25 ml three neck round bottomed flask. Then the solution was heated at 120 °C for 2 hour with nitrogen gas purging to remove water present in the acid. The solution was then collected under

hot condition in an airtight syringe and stored in a deaerated 30 ml vial fitting with a screw type septa. This stock solution was solidified at room temperature and for every reaction this stock solution was heated to 80°C and the desired amount of the melted stock solution was introduced in the reaction system.

Synthesis of $CsPbI_3$ Nanocubes. 92,2 mg of PbI_2 and 5 ml ODE were loaded in a 25 ml round bottomed flask and deaerated at 120 °C for 1 hour by purging nitrogen gas. Then 0.5 ml each of OLA and OA were injected into the reaction flask. 0,5 mL of pre-heated stock solution of OLA-HI was injected to the reaction system, and it was kept under heating condition for 10 minutes. Then the temperature was increased to 260 °C. Once the solution became clear, 0.5 ml Cs-oleate was injected swiftly. The reaction was stopped by removing heating mantel after 1 min of annealing and then it was allowed to cool down to room temperature naturally. The crude solution was taken in a centrifuge tube and centrifuged at 6000 rpm for 15 min at temperature of 17 °C. After centrifugation, the supernatant solution was discarded carefully and redispersed in hexane. To re-precipitate further, methyl acetate (hexane:methylacetate = 1:4) was added to the dispersed solution and again centrifuged at 6000 rpm for 2 min. The precipitate was redispersed in hexane.

DDAB Exchange in Solution. 1 mL of the pristine solution of $CsPbBr_3$ NCs was added to 0.3 mL of a 25 mM DDAB solution (in toluene) and the mixture was left stirring for 10 min at room temperature. Then the solution was washed with MeOAc and centrifuged at 4500 rpm for 5 minutes, then redispersed in toluene.

Thiocyanate Exchange in Solution. 1 mL of the pristine solution of $CsPbBr_3$ NCs was diluted with 3 mL of extra toluene and added to 20 mg of NH_4SCN. The mixture was left stirring for 2 hours due to the poor solubility of the thiocyanate in toluene. Then the solution was washed with MeOAc and centrifuged at 4500 rpm for 5 minutes, followed by dispersion in toluene.

$CsPbBr_3$ Film fabrication. Obtained NCs were left one night in the fridge (4°C) decanting. The supernatant was transferred in a separate vial. A counter-solvent to choose from acetonitrile, ethyl acetate or methyl acetate was added until the solution became turbid and the mixture centrifuged at 5000 rpm for 5 minutes. The precipitate was redispersed in the desired solvent (toluene, octane) varying the amount depending on the desired thickness of the film. The obtained clear solution was spin-coated onto a ITO/glass substrate at 1500 rpm for 40 seconds.

$CsPbI_3$ Film Fabrication. Four batches of NCs were mixed and left one night in the fridge (4°C) decanting. The surnatant was transferred in a separate vial. An additional centrifuge (1500 rpm – 4 minutes) was carried out and the obtained surnatant was spin-coated onto a ITO/glass substrate at 1500 rpm for 40 seconds.

Solid state ligand treatment and Layer-by-Layer deposition. For the DDAB ligand treatment, the $CsPbX_3$ layer was dynamically dropcast with a solution of 6 mM of DDAB in MeOAc/EtOAc (X=Br/I) and left spinning for 120 s at 1500 rpm. For the NH_4SCN ligand exchange analogue treatment was carried out with 10^{-4} mM of NH_4SCN in MeOAc/EtOAc (X=Br/I). Following layers were deposited with the same methodology. This procedure was repeated several times to achieve the desired thickness of the film. For instance, starting from a ~ 20 nm layer, 60 nm thick film was obtained with three LbL depositions or, starting from ~130 nm layer, 220 nm thick film was obtained with three LbL depositions. The film thicknesses were evaluated by AFM measurements.

Transmission Electron Microscopy. Bright field TEM images were acquired on a JEOL JEM-1011 microscope equipped with a thermionic gun at 100 kV accelerating voltage. The samples were prepared by drop-casting diluted $CsPbBr_3$ colloidal suspensions onto 200-mesh carbon-coated copper grids.

Photoluminescence quantum yield. PLQY measurements were carried out on pristine, ligand-treated NCs and multiple layer films with an Edinburgh Instruments fluorescence spectrometer (FLS920), which included a Xenon lamp with a monochromator for steady-state PL. The PL spectra recorded from films were obtained with an excitation wavelength of 400 nm. A calibrated integrating sphere was used to record the PLQY values from films on ITO/glass substrates.

Fourier Transform Infrared Spectroscopy. FTIR measurements were carried out in transmission mode with a Thermo Fisher 50 with a KBr Infrared Detector. Spectra were collected by averaging between 24 and 128 scans at 8 cm^{-1} resolution. Samples were prepared the same way as described in the $CsPbBr_3$ film fabrication but on substrates transparent in frequency range of 3800-1000 cm^{-1}.

UV−vis Absorption, PL. UV−visible absorption spectra were recorded using a Varian Cary 300 UV−vis absorption spectrophotometer (liquid sample) and Cary 5000.

AFM. Atomic force microscopy topographies were acquired using a XE-100 by Park Scientific, operating in non-contact mode. RMS roughness and film thickness values were computed from the statistical analysis of data by means of WsXM software.

Inductively Coupled Plasma (ICP-OES) Elemental Analysis. Elemental analyses were performed using an Inductively Coupled Plasm-Optical Emission Spectroscopy (ICP-OES) instrument (Thermo Fisher, iCap 6000). For sample preparation, 25µL of nanocrystal suspension were digested in 2.5 mL of Aqua Regia in a 25 mL volumetric flask. After one night of digestion at room temperature, flask was filled up with Milli-Q water and filtered through 0.2 µm PTFE membrane prior to the measurement.

SOLUTION PHASE LIGAND EXCHANGE OF $CsPbBr_3$ NANOCRYSTALS.

Solution phase ligand exchange was carried out according to the procedure reported in the Experimental Section. X-Ray diffraction pattern show a cubic phase for the treated sample as well as the pristine one (Figure S1 a,b,c). In Figure S1 d,e,f we report the size distributions for pristine, DDAB and NH_4SCN treated samples respectively. Abs/PL spectrum of DDAB and NH_4SCN treated samples present an excitonic peak at 504 nm (Figure S2a). PL emission of the NH_4SCN treated sample blue shifts by 3 nm to 508 nm (FWHM = 15 nm, Figure S2a) with respect to the pristine film. However, there is no equivalent change in the absorption spectrum and neither in

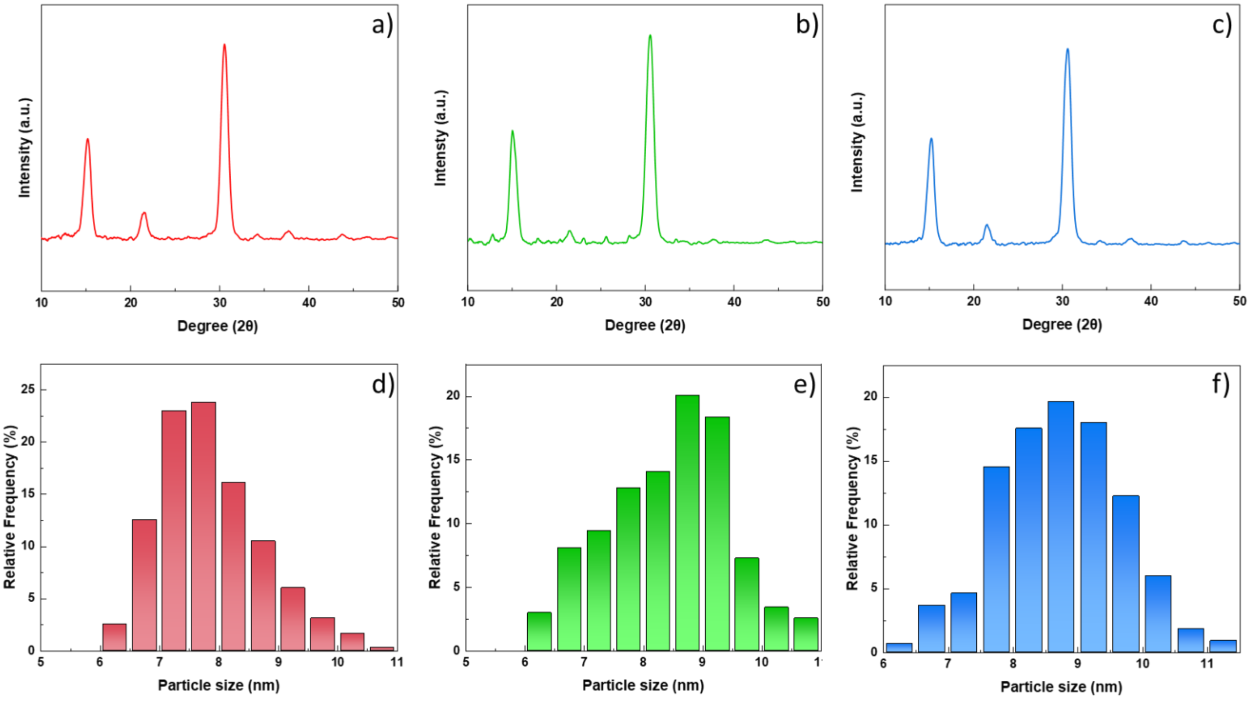

***S1**: XRD pattern of $CsPbBr_3$ colloidal nanocrystals untreated (a), DDAB treated (b) and NH_4SCN (c) treated in solution with relative size dispersion (d), (e), (f).*

XRD patterns, indicating that this shift is not caused by a decrease in size of the NCs, neither to a change in lattice parameters as already demonstrated by Wu et al,[1] thus suggesting a change in the emitting states as a consequence of ligand treatment, as suggested by Kosher at al.[2] On the other

hand, the PL of the DDAB treated sample does not present any shift compared to the pristine sample. TEM images of DDAB and NH4SCN treated samples are shown in Figure S2 b,c. The PLQY measured for the treated samples are 92 ± 8% for the DDAB and 40 ± 4% for NH_4SCN.

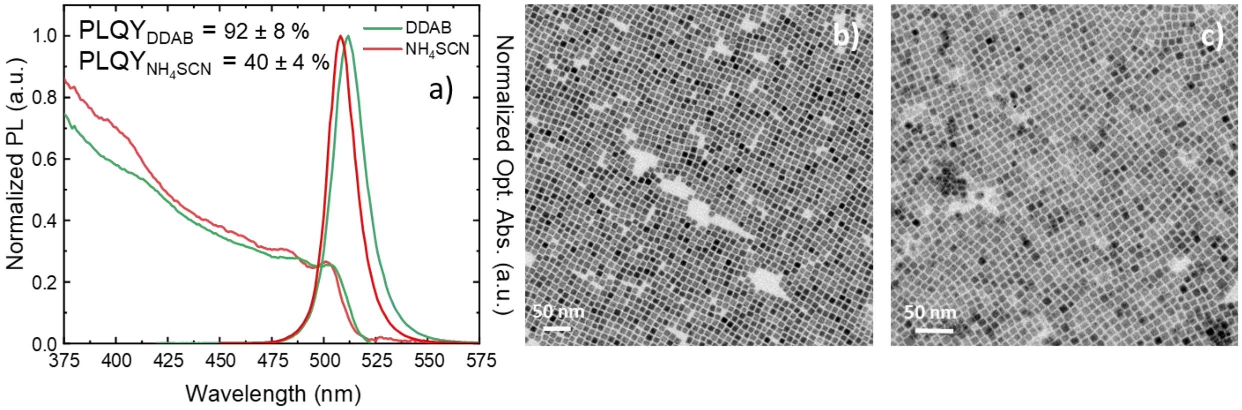

***S2**: a) Abs/PL spectra of solution-phase ligand treated $CsPbBr_3$ NCs. PLQY are referred to thin films fabricated after the treatment; b) TEM picture of solution-phase treated $CsPbBr_3$ nanocrystals with DDAB. c) TEM picture of solution-phase treated $CsPbBr_3$ nanocrystals with NH_4SCN.*

SOLID PHASE LIGAND EXCHANGE AND LAYER-BY-LAYER PROCEDURE.

A concentrated solution of $CsPbBr_3$ is obtained by mixing 2/3 batches of reported synthesis and leaving the obtained solution in the fridge (4°C) for one night. For an optimal result, once taken the supernatant, this procedure is repeated twice. A variable amount of counter-solvent (preferably ethyl acetate or acetonitrile) is added until the solution becomes turbid. After that, it is centrifuged at 5000 rpm for 6 minutes and redispersed in 400 µL of toluene.

A variable amount of obtained solution (depending on substrate dimension) is deposited on ITO/glass and spin-coated at 1500 rpm for 2 minutes. For DDAB treatment, 6 mM DDAB solution in methyl acetate is drop-cast on the nanocrystal film and left spinning for 2 minutes. Same procedure for 0.01 mM of NH_4SCN solution in methyl acetate. From now on, it is possible to deposit again the concentrated solution on $CsPbBr_3$ and repeat the ligand treatment 3/4 times until the desired film thickness is obtained. Optical density measured at every step of deposition are reported in Figure S3 a and b respectively for the DDAB and NH_4SCN treatment.

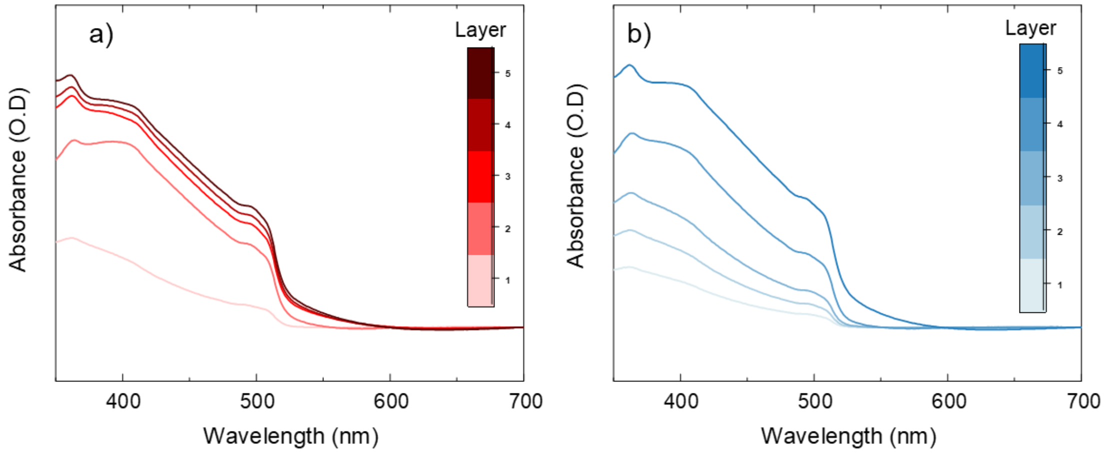

S3: *Optical density spectra recorded with increasing number of layers deposited following Layer-by-Layer assembly for DDAB (a) and* NH_4SCN *(b).*

ATOMIC FORCE MICROSCOPY CHARACTERIZATION

We acquired root mean square (RMS) roughness and film thickness values for all the samples built with the LbL method, after each layer growth step and as a function of the solvent used for the solid-state ligand treatment. Measurements were systematically gathered along different sample coordinates and averaged (see Table S1).

	Layer 1 thickness (nm)	Layer 2 thickness (nm)	Layer 3 thickness (nm)	Layer 1 RMS (nm)	Layer 2 RMS (nm)	Layer 3 RMS (nm)
$CsPbBr_3$ (DDAB)	126	157	385	7	9	11
$CsPbBr_3$ (NH_4SCN)	130	160	220	25	21	28
$CsPbI_3$ (DDAB)	76	131	202	8	7	11
$CsPbI_3$ (NH_4SCN)	40	78	127	7	10	16

Table S1: *Average values of RMS roughness and film thickness as a function of number of layers for DDAB and NH_4SCN treated films*

In Fig. S4 we show a typical AFM image of a scratch performed on a sample, used to evaluate the film thickness.

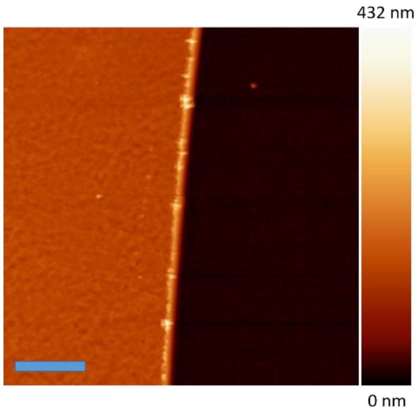

S4*: AFM topography of a scratched, 3 layers thick CsPbBr3 nanocrystal film treated with DDAB. The blue scale bar is 6 µm long.*

$CsPbI_3$ NANOCRYSTALS LAYER-BY-LAYER ASSEMBLY

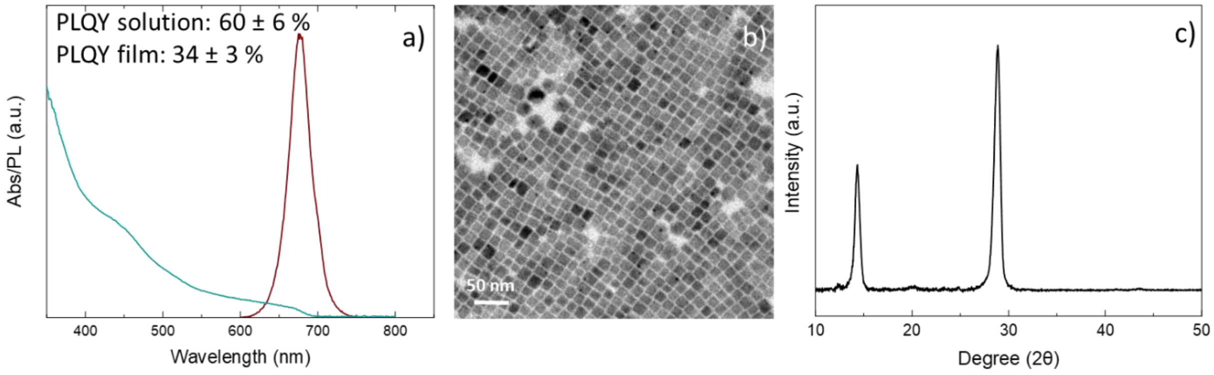

***S5**: (a) Abs/PL/PLQY properties of $CsPbI_3$ colloidal solution and film PLQY, (b) TEM picture of colloidal $CsPbI_3$, nanocrystals (c) XRD patter of CsPbI3 nanocrystals,*

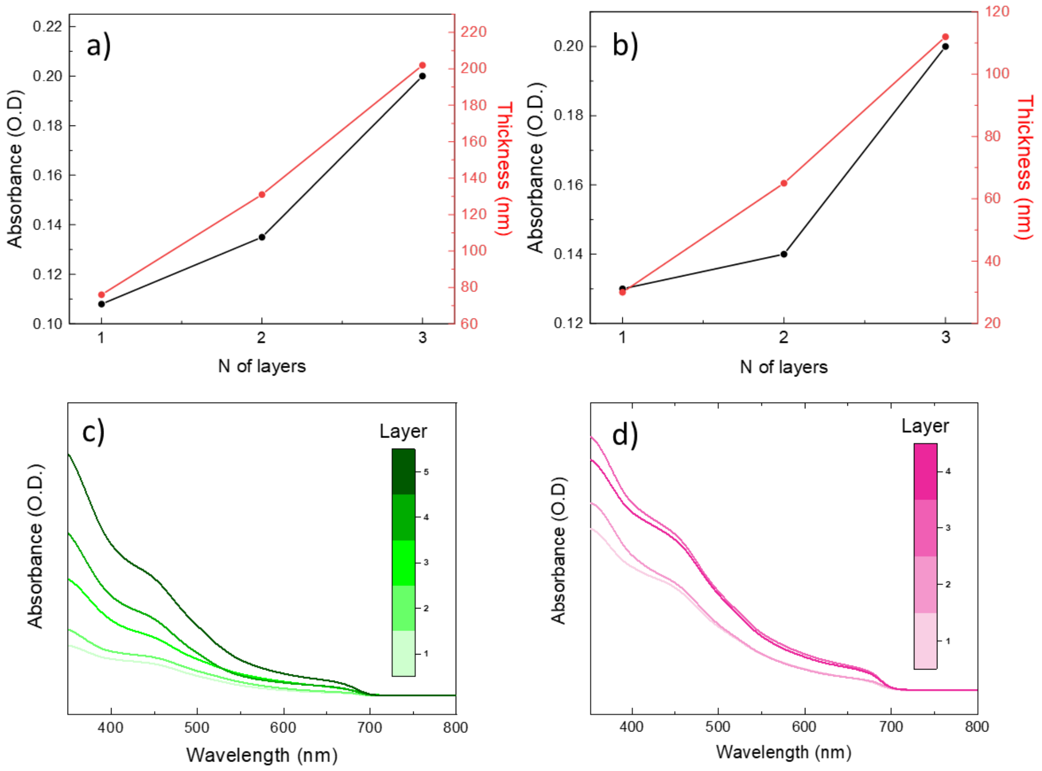

***S6**: (a) Optical density at 500 nm for DDAB treated samples of $CsPbI_3$ at increasing number of layer and relative thickness. (b) Optical density at 500 nm for SCN treated samples of $CsPbI_3$ at increasing number of layer and relative thickness. (c) Optical density spectra recorded at increasing number of layer for CsPbI3 NCs treated with DDAB and NH_4SCN (d).*

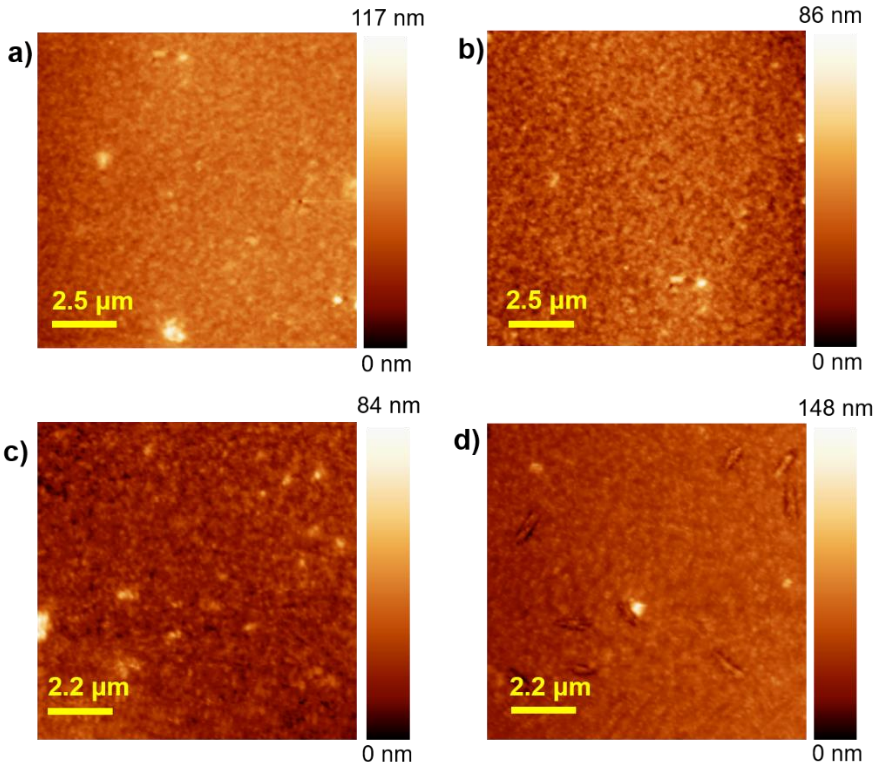

***S7**: (a, b) AFM topography of 1-layer films treated with DDAB and NH_4SCN, respectively. (c,d) AFM topography of 2-layers films treated DDAB and NH_4SCN, respectively.*
